# P-1546. Clinical Characteristics and Treatment Outcomes of Isoniazid-Resistant, Rifampicin-Susceptible Tuberculosis Admitted to a National University Hospital in the Philippines

**DOI:** 10.1093/ofid/ofaf695.1726

**Published:** 2026-01-11

**Authors:** Jerome G Manzano, Lerma Bhelle B Iglesia, Vikki Darl R Rodriguez, Julius Patrick R Miranda, Regina Berba

**Affiliations:** University of the Philippines- Philippine General Hospital/ Philippine Society for Microbiology and Infectious Diseases/ Dr. Paulino J. Garcia Memorial Research and Medical Center, Cabanatuan City, Nueva Ecija, Philippines; University of the Philippines- Philippine General Hospital/ Philippine Society for Microbiology and Infectious Diseases, Manila, La Union, Philippines; University of the Philippines- Philippine General Hospital/ Philippine Society for Microbiology and Infectious Diseases, Manila, La Union, Philippines; University of the Philippines- Philippine General Hospital, Tagum, Rizal, Philippines; University of the Philippines Manila, Manila, National Capital Region, Philippines

## Abstract

**Background:**

Tuberculosis is a disease that is regarded to be of global health importance. Isoniazid is used in short course second line treatment for drug resistant tuberculosis. It is not only needed for the treatment of active tuberculosis but also highly effective in preventing disease and for latent TB infection. However, isoniazid resistance is regarded to undermine the treatment for TB disease and infection.

**Figure:**
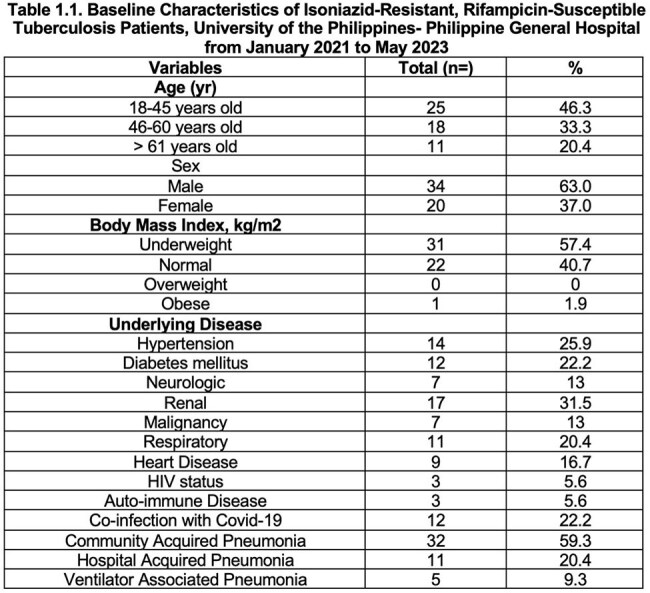

**Figure:**
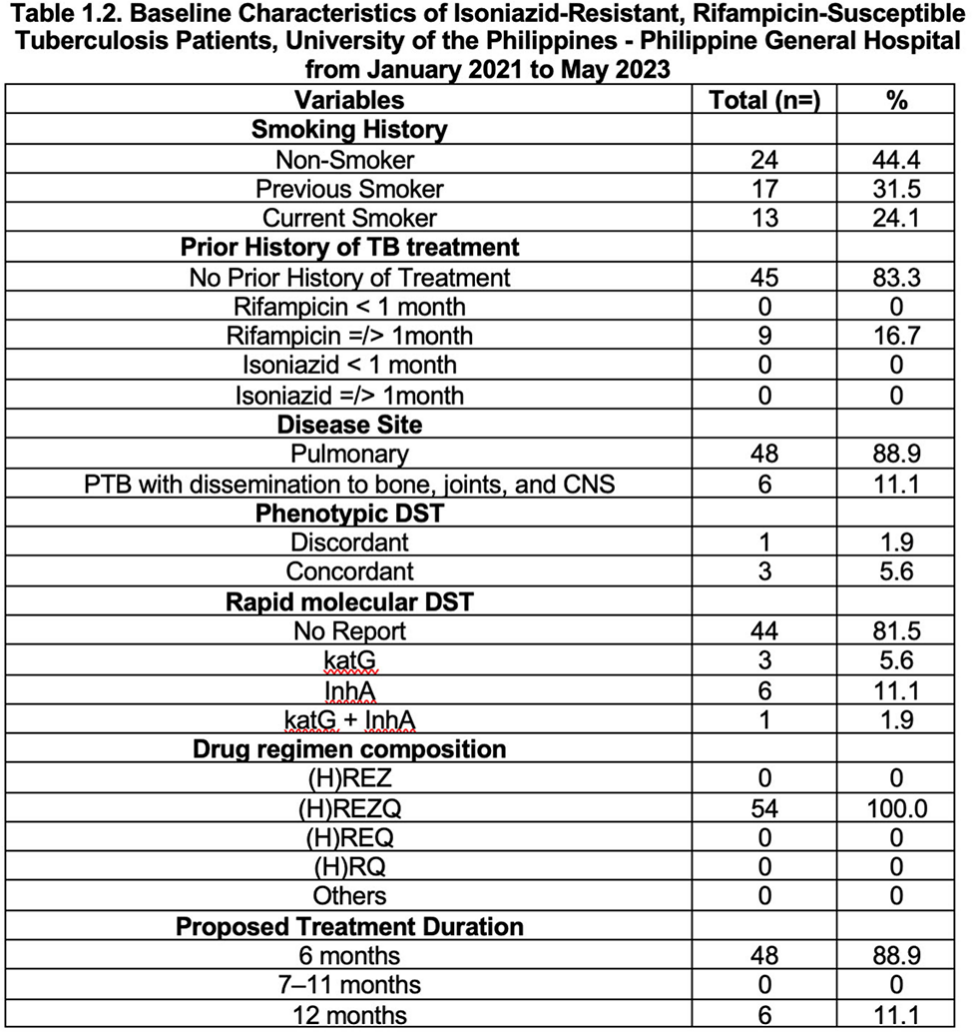

**Methods:**

Retrospective research design was used in the study. The study was conducted at University of the Philippines- Philippine General Hospital. The investigators reviewed the electronic records of patients who were diagnosed to have active, isoniazid-resistant, rifampicin-susceptible Tuberculosis during their admission to PGH from January 2021 to May 2023. The investigators initially identified patients through the TB results logbook at the Central Lab. The case numbers were taken and were submitted to Medical records for chart retrieval. Patient data were collected from electronic medical records and it included demographic features, as well as results of laboratory, radiographic, and microbiological tests.

**Figure:**
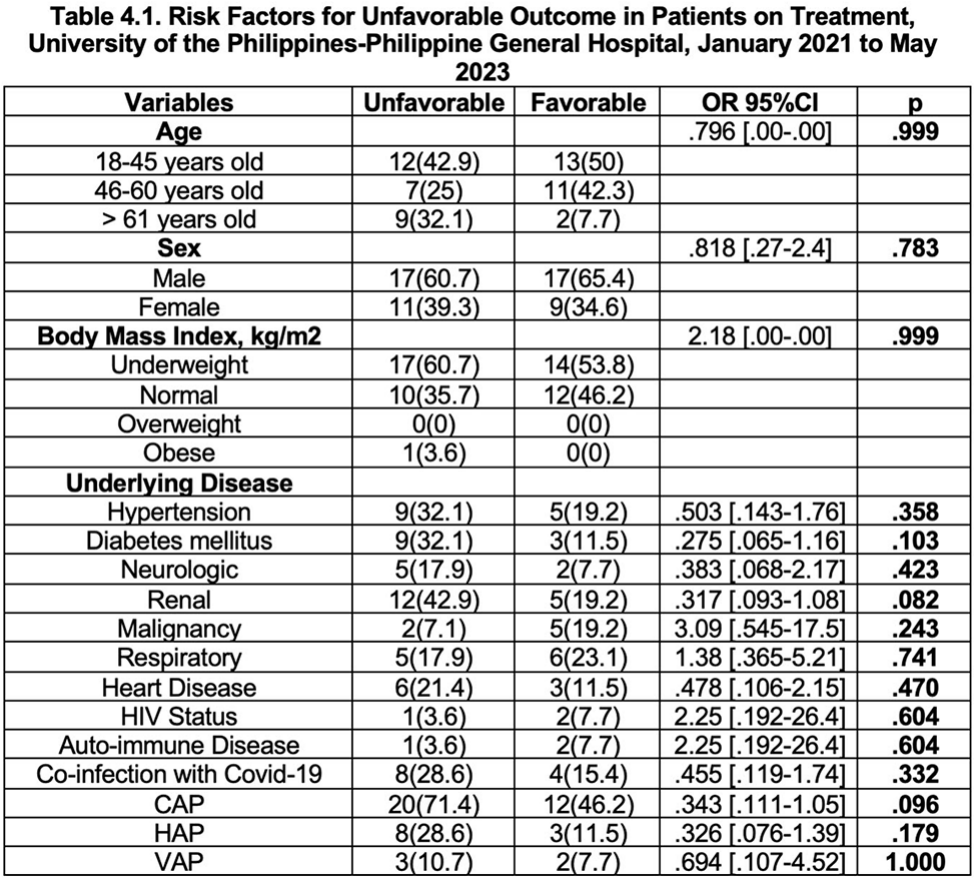

**Figure:**
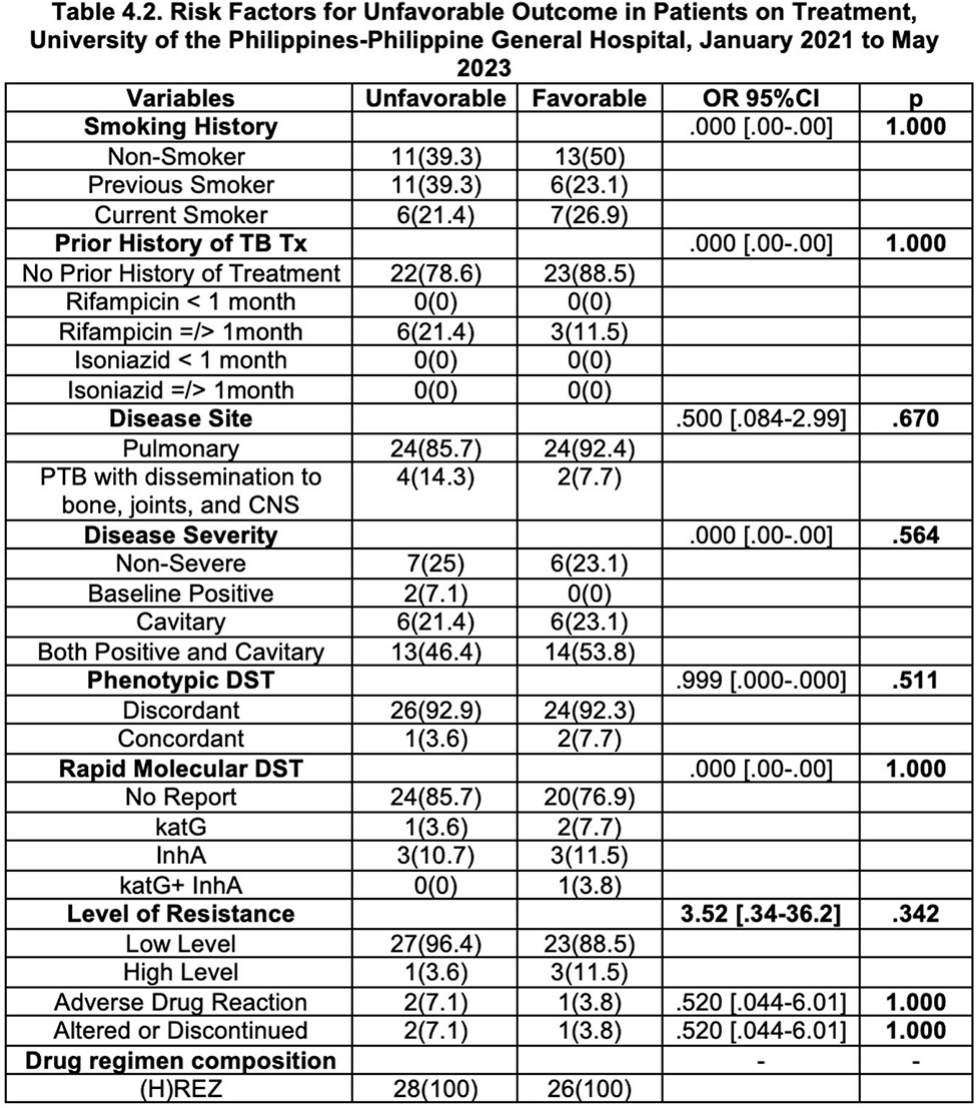

**Results:**

Patients with Isoniazid-resistant, Rifampicin susceptible tuberculosis were mostly in the 18 to 45 years of age, male, and were underweight. The majority of these patients had no adverse drug reaction and half of these patients were discharged and another half of these cases had poor outcome.

**Conclusion:**

The study revealed that isoniazid resistance among these patients is not caused by previous intake of anti-TB drugs. Rather, the pattern of resistance is a primary drug resistance suggesting that they were infected from exposure to isoniazid-resistant index cases. Considering the limited access of Filipino patients to rapid diagnostic tests that detect TB gene mutations, isoniazid resistance in rifampicin-susceptible patients could be unrecognized. More dedicated studies should be done to explore the following: a) the effect of isoniazid monotherapy in the emergence of isoniazid resistance, b) the mechanism and pattern or resistance among patients with isoniazid-resistant, rifampicin-susceptible tuberculosis, and most importantly, and c) the prevalence of TB preventive therapy (using isoniazid) failure.

**Disclosures:**

All Authors: No reported disclosures

